# Relevance of Extending FGFR3 Gene Analysis in Osteochondrodysplasia to Non-Coding Sequences: A Case Report

**DOI:** 10.3390/genes15020225

**Published:** 2024-02-10

**Authors:** Zangbéwendé Guy Ouedraogo, Caroline Janel, Alexandre Janin, Gilles Millat, Sarah Langlais, Bénédicte Pontier, Marie Biard, Mathis Lepage, Christine Francannet, Fanny Laffargue, Isabelle Creveaux

**Affiliations:** 1 Service de Biochimie et Génétique Moléculaire, CHU Gabriel Montpied, CHU Clermont-Ferrand, 63000 Clermont-Ferrand, France; cnachury@chu-clermontferrand.fr (C.J.); slanglais@chu-clermontferrand.fr (S.L.); mlepage@chu-clermontferrand.fr (M.L.); 2 Université Clermont Auvergne, CNRS, Inserm, iGReD, 63001 Clermont-Ferrand, France; 3 Unité Fonctionnelle Cardiogénétique, Moléculaire, Centre de Biologie et Pathologie Est, Hospices Civils de Lyon, 69677 Bron, France; alexandre.janin01@chu-lyon.fr (A.J.); gilles.millat@chu-lyon.fr (G.M.); 4 CNRS UMR5261, INSERM U1315, Pathophysiology and Genetics of Neuron and Muscle, Institut Neuromyogène, Université Claude Bernard Lyon 1, 69008 Lyon, France; 5 Service de Génétique Médicale, CHU Estaing, CHU Clermont-Ferrand, 63100 Clermont-Ferrand, France; bpontier@chu-clermontferrand.fr (B.P.); cfrancannet@chu-clermontferrand.fr (C.F.); flaffargue@chu-clermontferrand.fr (F.L.); 6 Service de Radiologie Pédiatrique, CHU Estaing, CHU Clermont-Ferrand, 63100 Clermont-Ferrand, France; mbiard@chu-clermontferrand.fr

**Keywords:** osteochondrodysplasia, skeletal dysplasia, achondroplasia, hypochondroplasia, fibroblast growth factor receptor 3, exonization

## Abstract

Skeletal dysplasia, also called osteochondrodysplasia, is a category of disorders affecting bone development and children’s growth. Up to 552 genes, including fibroblast growth factor receptor 3 (*FGFR3*), have been implicated by pathogenic variations in its genesis. Frequently identified causal mutations in osteochondrodysplasia arise in the coding sequences of the *FGFR3* gene: c.1138G>A and c.1138G>C in achondroplasia and c.1620C>A and c.1620C>G in hypochondroplasia. However, in some cases, the diagnostic investigations undertaken thus far have failed to identify the causal anomaly, which strengthens the relevance of the diagnostic strategies being further refined. We observed a Caucasian adult with clinical and radiographic features of achondroplasia, with no common pathogenic variant. Exome sequencing detected an *FGFR3*(NM_000142.4):c.1075+95C>G heterozygous intronic variation. In vitro studies showed that this variant results in the aberrant exonization of a 90-nucleotide 5′ segment of intron 8, resulting in the substitution of the alanine (Ala359) for a glycine (Gly) and the in-frame insertion of 30 amino acids. This change may alter FGFR3’s function. Our report provides the first clinical description of an adult carrying this variant, which completes the phenotype description previously provided in children and confirms the recurrence, the autosomal-dominant pathogenicity, and the diagnostic relevance of this *FGFR3* intronic variant. We support its inclusion in routinely used diagnostic tests for osteochondrodysplasia. This may increase the detection rate of causal variants and therefore could have a positive impact on patient management. Finally, *FGFR3* alteration via non-coding sequence exonization should be considered a recurrent disease mechanism to be taken into account for new drug design and clinical trial strategies.

## 1. Introduction

Skeletal dysplasia, also called osteochondrodysplasia, refers to a group of skeletal disorders resulting from abnormalities in bone development and growth [1,2]. Up to 771 distinct entities have been identified and classified into 41 groups in the 11th updated nosology of genetic skeletal disorders established by the Nosology Group of the International Skeletal Dysplasia Society (ISDS) [3]. Its global incidence is estimated at 1:5000 births [1,4]. The number of identified genes reached 552 in the 2023 revised nosology [3]. These genes were functionally diverse, implicated in a broad range of biological processes, and were altered by a variety of mutational mechanisms [5]. The molecular characterization of osteochondrodysplasia revealed that pathogenic variants of the same gene, such as *FGFR3* (fibroblast growth factor receptor 3), can trigger strikingly different phenotypes (phenotypic heterogeneity), and the same phenotype can result from different alterations in different genes (genetic heterogeneity) [5].

Based on several features, including molecular criteria, *FGFR3*-related skeletal dysplasia phenotypes are defined into groups 1, 30, 33 and 40 of the nosology. Except in a few cases of Camptodactyly, tall stature, and hearing loss syndrome (CATSHLS, MIM phenotype number # 610474) [6], most disease-causing *FGFR3* germline alterations are autosomal-dominant mutations. The mechanism of disease causation is a gain of function, usually associated with missense mutations or delins [7,8]. The most frequently involved mutations are, in achondroplasia, substitutions triggering a p.Gly380Arg change in the FGFR3 protein (c.1138G>A and c.1138G>C) [9,10], and in hypochondroplasia, p.Asn540Lys (c.1620C>A and c.1620C>G) [9,11].

Because of its phenotypic and genetic heterogeneity, the molecular diagnosis of osteochondrodysplasia remains a challenge. The usual strategies include performing targeted analysis of frequent mutations or phenotype-based panel sequencing, looking for previously identified mutations in the origin population of the patient [12]. However, the performance of phenotype-based panel sequencing depends on the completeness of the panels. Moreover, most pathogenic variants have not been reported at a high enough frequency to allow the establishment of genotype/phenotype correlations, so the prognosis is sometimes difficult to discern.

Here, we report the first identification in a Caucasian patient with osteochondrodysplasia of a pathogenic intronic variant of *FGFR3*. Naturally, the usual osteochondrodysplasia panel-based sequencing strategy failed to identify the pathogenic variant, as it was an intronic cryptic splicing variant located far from the exonic sequences, at a base position not usually involved in variant calling during ordinary panels or exome analyses. In fact, such analyses only include exons and a limited number (5–30) of non-coding nucleotides (exon-intron junctions) flanking both sides of each exon. The variant’s effect on the *FGFR3* gene expression was characterized using minigene assay. While the minigene analyses were being performed, this variant was reported as likely pathogenic in three patients who were Chinese children with skeletal dysplasia [7]. Our report provides the first clinical description of an adult patient carrying the *FGFR3*:c.1075+95C>G (GRCh37-chr4:g.1805658C>G) variant and strengthens the relevance of including this recurrent variant in the genetic testing of patients with osteochondrodysplasia or clinical presentation matching *FGFR3* alteration. Finally, this variant leading to the exonization of an intronic sequence is here confirmed as a recurrent disease mechanism to be considered in further exploration for molecular diagnosis and also drug design and clinical trials.

## 2. Materials and Methods

### 2.1. Next-Generation Sequencing (NGS)

Whole-exome sequencing (WES) high-throughput paired-end sequencing was performed using the NextSeq 500 platform (Illumina^®^, San Diego, CA, USA) and the NextSeq 500 high-throughput kit v2.5 (Illumina^®^, San Diego, CA, USA) for 150 cycles. Prior to sequencing, the exomes were captured and enriched using the Human Core Exome + RefSeq panel (Comprehensive Exome, Twist Biosciences^®^, South San Francisco, CA, USA). The sequencing data were analyzed using the SeqOne Genomics platform for read alignment, variant calling, and annotation (SeqOne Genomics^®^, Montpellier, France). The variants were filtered using the following parameters: quality score ≥ 20, depth ≥ 5×, and presence in ≥20% of reads. The variants were interpreted and classified according to the joint consensus recommendation of the American College of Medical Genetics and Genomics and the Association for Molecular Pathology [13].

### 2.2. Sanger Sequencing

The genomic DNA was extracted from the patient’s peripheral blood sample for *FGFR3* gene sequencing. The target DNA segment was amplified using specific DNA primers. The primer sequences are available on request. The amplified sequences were fluorescently labeled using the BigDye Terminator v1.1^®^ cycle sequencing kit (Applied Biosystems, Waltham, MA, USA). The labeled DNA fragments were then purified. After electrophoresis using a capillary 3130xl genetic analyzer (Applied Biosystems, Waltham, MA, USA), the final analysis was performed using the sequencing analysis software SeqScape v3 (Applied Biosystems, Waltham, MA, USA).

### 2.3. Minigene Assay

The minigene assay was performed according to the previously reported method [14] to determine the variant’s effect on *FGFR3* transcript splicing. Briefly, a 699 bp fragment, consisting of 185 bp IVS7 + 145 bp exon 8 + 369 bp IVS8, was cloned using PCR from the genomic DNA of the patient with the aid of Phusion^®^ High-Fidelity DNA Polymerase (New England Biolabs, Ipswich, MA, USA). Notice that he was heterozygous in this substitution. The cloned sequences were inserted into the *NdeI* cloning site of the minigene vector pTB using the In-Fusion HD PCR Cloning Kit (Takara Bio, Kusatsu, Japan) [15]. Then, the quality of the cloning, mainly the presence of c.1075+95C>G in the targeted intronic sequence of the mutant, was confirmed using sequencing. Furthermore, a normal clone and a mutant clone were transfected into HeLa cells. The HeLa cells were seeded at a concentration of 5 × 10^4^ cells per well in a 12-well cluster plate in 1 mL of medium and transiently transfected with 1 μg of plasmid using FuGENE (Promega, Madison, Wisconsin) following the manufacturer’s recommendations. Then, 48 hours after transfection, total RNA was extracted using RNAqueous-4PCR Total RNA Isolation Kit (Thermo Fisher, Waltham, MA, USA) and then treated using DNase (DNA-free DNA Removal Kit, Thermo Fisher, Waltham, MA, USA). RT-PCR was performed using the Transcriptor High Fidelity cDNA Synthesis Kit (Roche Molecular Diagnostics, Indianapolis, Indiana) and using 250 ng of total RNA and random primers. cDNA amplification was performed using vector-specific primers surrounding the cloning site and HotStarTaq Plus DNA Polymerase (Qiagen, Hilden, Germany). The PCR products were resolved on 2% agarose gel and sequenced to identify the splicing events. All the transfection experiments were performed in triplicate.

## 3. Results

### 3.1. Patient Clinical History and Phenotype

**Figure 1:**
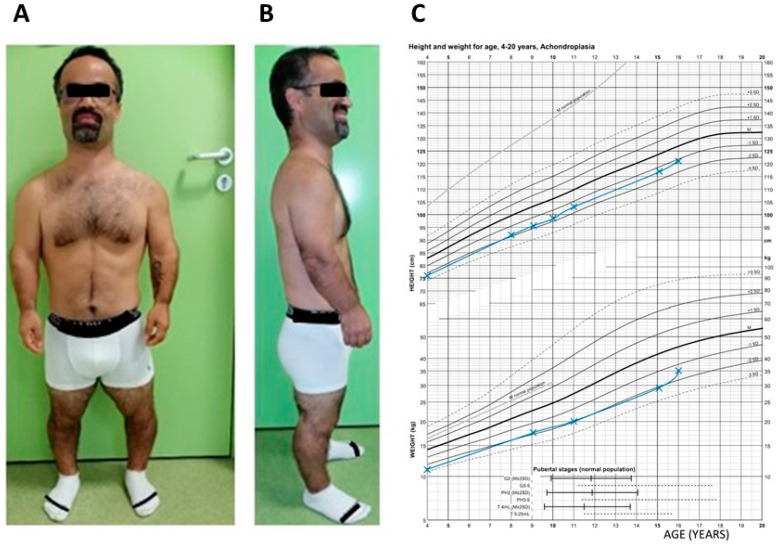
Clinical features of the proband. (**A**) Frontal and (**B**) profile pictures of the patient presenting a stocky build, short stature, a bossing forehead, shortening of the limb segments, small hands and feet, and a bilateral genu varum. (**C**) Growth charts (height and weight) built in blue lines with achondroplasia growth chart reference template [16].

A 42-year-old man was referred to the genetics department for etiologic diagnosis for unspecified osteochondrodysplasia. He presented a stocky build and a short stature of 131 cm (<−4 DS), with an average head circumference (57 cm, +0.5 DS) and weighing 47 kg (−3 DS). (Figure 1). His upper body segment was measured as 84 cm and his arm span as 124 cm. He had a bossing forehead. A preliminary physical examination found an ogival palate, a shortening of the proximal and middle limb segments with small hands and feet, a bilateral genu varum, and lumbar scoliosis. His past medical history reveals that he was born of a normal pregnancy with the following anthropometric measurements: a 48 cm (−1 DS) length, 34 cm (−1 DS) head circumference, and 2700 g (−2 DS) weight. A bossing forehead and micromelia were also obvious. A continuous growth retardation was recorded. His growth curves are recorded on a reference template of achondroplasia growth charts in Figure 1C. [16].

**Figure 2:**
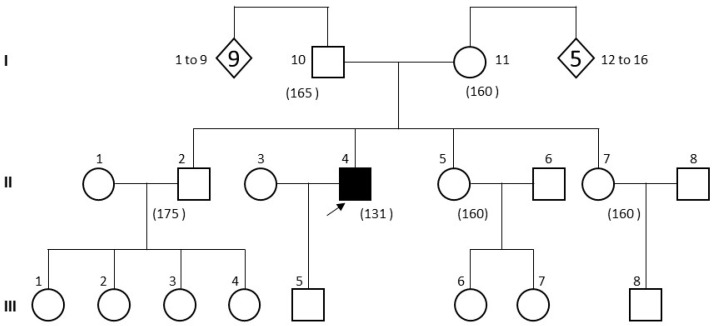
The patient family pedigree. Male II-4 is the proband, presenting with skeletal dysplasia. Subjects’ height (in cm) is indicated in brackets.

He had developed normal intelligence, often complaining of paresthesia in the right upper limb due to a C5–C6 cervical radiculopathy and of spinal pain related to the scoliosis. He was being followed up for bilateral myopia. No inbreeding or any other case of osteochondrodysplasia was recorded in his pedigree (Figure 2).

**Figure 3:**
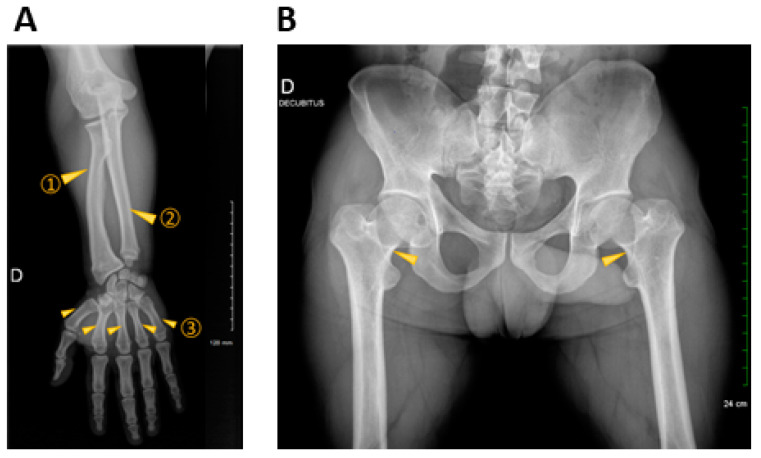
X-ray findings in proband. (**A**) A standard radiograph of right forearm and hand with shortening of the radius ①, the ulna ②, and the metacarpal bones ③. (**B**) Standard radiograph of pelvis showing a major shortening of the femoral necks (arrowheads). D: right.

Repeated medical imaging, including standard radiographs (Figure 3), computed tomography, and magnetic resonance imaging, confirmed the micromelia and the cervical radiculopathy. They found an aspect of a narrowed lumbar canal staggered from L1 to S1 with a hypertrophy of the pedicle in L5–S1 and the left posterior articulation pushing back on the dural sac to the right. They also revealed a lumbosacral discopathy in L2-L3 and L3-L4 right lateral foraminal stenosis. The biochemical estimation of his urinary glycosaminoglycans was quite normal, including the electropherograms. Early genetic analyses, including comparative genomic hybridization, targeted analysis of the two common pathogenic variants in achondroplasia, and osteochondrodysplasia panel-based NGS (49 genes including *FGFR3*), did not identify any causal anomaly.

### 3.2. Variant Discovery and Analysis

**Figure 4:**
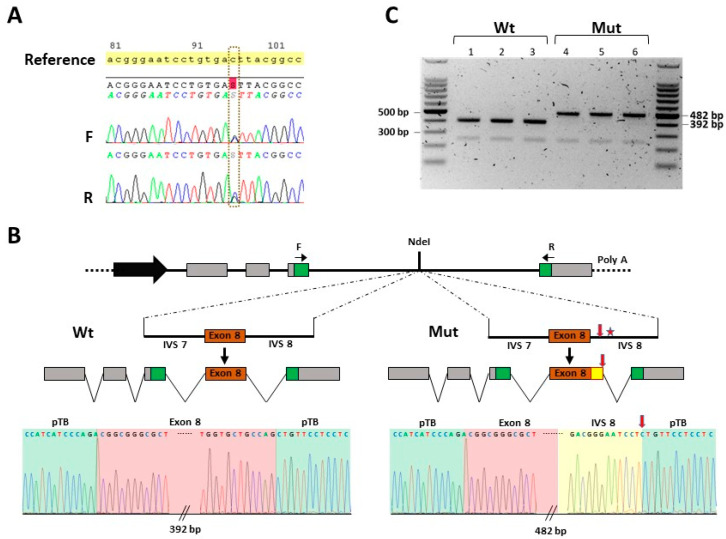
Confirmation of pathogenicity of *FGFR3*:c.1075+95C>G heterozygous intronic transversion. (**A**) Sanger sequencing data showing *FGFR3*:c.1075+95C>G heterozygous intronic variant. (**B**) Design of minigene assay displaying plasmid construct, wild-type (Wt), and mutant (Mut) inserts. Each insert consisted of a fragment of 699 bp, made up of 185 bp IVS7 + 145 bp exon 8 + 369 bp IVS8. The c.1075+95C>G variant is indicated as a red star and the cryptic splice donor site (indicated by vertical red arrow) activated at five nucleotides upstream of the mutation. Normal splicing resulted from Wt clones and aberrant splicing from mutants. In abnormal splicing, the 5′ end 90 nucleotides of IVS8 are retained as a coding sequence adjacent to exon 8 (see yellow box). Sequencing results confirming normal (Wt) and aberrant splicing (Mut) are displayed in the lower panel. The exonized sequence is highlighted in yellow, that of exon 8 in light red, and the plasmid sequence in light green. Black horizontal arrows represent PCR primers and black vertical arrows point to the representation of the splicing of the primary transcripts. (**C**) Picture of electrophoresis gel of RT-PCR products of RNA extracted from cells transfected with Wt or Mut vectors. Normal splicing products 392 bp in size are detected in cells transfected with wild-type vectors in triplicate (Wt), and abnormal splicing products 482 bp in size were generated from the mutant sequences (Mut). Bp: base pairs; F: forward; IVS: intron; Mut: mutant; pTB: plasmid; R: reverse; Wt: wild-type.

We performed exome sequencing on the genomic DNA of the patient and his genitors. The initial analysis included exons and exon–intron junctions flanking both sides of each exon. Variants were filtered for a quality score ≥ 20, depth ≥ 5×, and presence in ≥ 20% of reads. It did not detect any relevant variant. Since the presentation was highly suggestive of a typical achondroplasia case, we extended the variant calling to all sequenced segments of *FGFR3,* i.e., no longer limited to exons and junctions. The *FGFR3*(NM_000142.4):c.1075+95C>G substitution variant was detected as a heterozygous de novo intronic variant in the proband since it was absent in both parents. This transversion was relevantly predicted using SpliceAI (https://spliceailookup.broadinstitute.org/) (30 June 2020) and some other computational prediction tools to be susceptible to altering the splicing of the *FGFR3* primary transcript by activating a cryptic splice donor site five bases upstream of the variant. The variant was not found among the genomes in gnomAD (v4) despite a good mean coverage. It was also absent from other control databases, including 1000 Genomes (30 June 2020) and dbSNP (v156). Moreover, a c.1075+30G>C single-nucleotide polymorphism (SNP) paternally inherited was detected in *cis* with the c.1075+95C>G variant. The NGS results were confirmed using Sanger sequencing (Figure 4A) and multiplex amplification of short tandem repeats.

The minigene assay (Figure 4B,C) was performed to elicit the functional effect of this variant on the *FGFR3* primary transcript splicing. The wild-type construct generated a major product at 392 bp corresponding to normal transcripts (Figure 4B,C). The results obtained with the construction carrying the c.1075+95C>G *FGFR3* variant exhibited a full effect on the splicing. It produced a major product at 482 bp (on the scale) corresponding to an exonization of the subsequent 90 nucleotides of the 5′ end of intron 8 adjacent to exon 8 (Figure 4B,C). This exonization did not induce a stop codon. However, it would trigger a deletion of the amino acid Ala359 and an insertion of 31 amino acids into the FGFR3 protein. We also detected a very low proportion of correctly spliced transcripts.

The damaging effects sustained the classification of this variant as pathogenic (Table 1) and its submission to the MobiDetails and LOVD^3^ databases (see data availability).

The variant met three criteria of pathogenicity justifying its classification as a pathogenic variant: PM2 is a criterion of moderate evidence of pathogenicity, PS2 of strong evidence, and PS3 of very strong evidence.

Integrating all of the results allowed a description of the subsequent change in the FGFR3 protein primary structure. The c.1075+30G>C SNP would trigger the substitution of a corresponding alanine (A) residue with a proline (P). This would result in a p.(Ala359delinsGlyThrGlyPheCysCysCysCysCysSerProLeuSerGlyGlyArgTrpLeuGlyThrArgGluSerCysGluAspGlyArgGluSerSer) change in the extracellular juxtamembrane region of the receptor at the protein level.

## 4. Discussion

The clinical features of this patient included a short stature, facial dysmorphism (restricted to frontal bossing), a stocky build, rhizomelia, mesomelia, short hands and feet, genu varum, and cervical radiculopathy. The radiologic findings consisted of multiple spinal abnormalities. Despite the acceptable controversy, such a clinical presentation and the a posteriori identification of a pathogenic *FGFR3* variant suggested the diagnosis of achondroplasia or severe hypochondroplasia, although the phenotype lacked some criteria, such as macrocephaly. Clearly, distinguishing between achondroplasia and hypochondroplasia is not easy even after the identification of a pathogenic *FGFR3* variant because of their overlapping features. We finally confirmed the diagnosis of achondroplasia or severe hypochondroplasia, as recently carried out for a carrier of this variant [7]. However, the phenotype/genotype correlation remains impertinent because of the small number of subjects, the missing description of adulthood in the previous (pediatric) cases, and the lack of some childhood information in our current case. Nevertheless, a comparative description is provided in Table 2.

Minigene assays confirmed the in silico prediction of the variant’s damaging effect on the *FGFR3* transcript splicing, by means of a construct different from that of the previous report, which used full sequences of exon 8, intron 8, and exon 9. Despite such a difference, the results were consistent, confirming the per se pathogenicity of the transversion. Recently (2 December 2023), the variant was reported in ClinVar as a pathogenic variation associated with hypochondroplasia or achondroplasia, thus augmenting the number of affected carriers to five. The predicted change in the FGFR3 protein is a substitution of Ala359 for a glycine (Gly) and an insertion of 30 amino acids into a segment adjacent to the receptor transmembrane domain containing the amino acid Gly380, involved in the most recurrent pathogenic variations found in achondroplasia. This predicted delins is also near one of the three immunoglobulin-like domains of the receptor [7], where the insertion of Ser-Phe after the position Leu324 consecutive to a de novo 6 base pair tandem duplication in the *FGFR3* gene was previously reported as a disease-causing mechanism in a patient with achondroplasia [17]. This insertion was shown in functional analyses to induce aberrant dimerization, excessive spontaneous phosphorylation of the FGFR3 dimers, and excessive ligand-independent tyrosine kinase activity, meaning pathologic hyperactivation of the FGFR3 pathway, which is the common pathophysiological mechanism of achondroplasia (gain of function) [17]. The exonized sequence also introduces six cysteine residues that could be engaged in intra-chain and inter-chain disulfide bridge formation, which can deregulate the receptor activation, as described with other proteins. In addition, the introduction of four serine residues may cause receptor dysfunction by activating or inhibiting serine phosphorylation. Even though this list is not exhaustive, we are able to speculate that they may explain how the delins we describe in our patient can disturb the receptor transmembrane domain, the adjacent immunoglobulin-like domain, or the protein thermodynamics and induce aberrant hyperactivated downstream signals, causing achondroplasia. Further studies should explore the consequences of this variant on the FGFR3 protein structure, conformation, dimerization, and signal transduction. In vivo studies may help highlight the variant’s developmental effect. Note that only de novo cases have been reported. Moreover, our patient successfully fathered a healthy son without any medical assistance. However, in genetic counseling, for a heterozygously affected individual with a partner of average stature, such observations do not modify the theoretical 50% risk in each pregnancy of having a child with achondroplasia.

## 5. Conclusions

In summary, we detected the *FGFR3*(NM_000142.4):c.1075+95C>G pathogenic variant in a heterozygous state in a Caucasian adult with achondroplasia. We provided a phenotype description that may help further genotype/phenotype correlation or prognosis evaluation in childhood patients or in prenatal cases. Concordantly with the first report of this variant, our report strengthened the relevance of refining the routinely used diagnostic strategies for osteochondrodysplasia by including this recurrent variation in targets to improve patient management. We moreover suggest that the research on osteochondrodysplasia’s causal mutations should be extended to the entire *FGFR3* gene when initial investigations do not find an alteration but the phenotype matches since molecular diagnosis impacts the design and testing of new FGFR-based therapies. This report confirms the exonization of this intronic sequence as a new disease mechanism to be considered in therapeutic strategy design.

## Figures and Tables

**Table 1:** Provided evidence for classifying *FGFR3*(NM_000142.4):c.1075+95C>G.

Criteria	PM2	PS2	PS3
**Evidence**	The variant is absent from control databases, including gnomAD, despite good coverage of the genomic region in sequencing.	The variant is confirmed to be de novo using NGS and Sanger sequencing, with both maternity and paternity confirmation, in a patient with the disorder and no family history of this trouble.	The minigene in vitro functional analyses confirmed, in two independent studies, the damaging effect on *FGFR3* transcript splicing.

**Table 2:** Summary of phenotype description of patients with *FGFR3*(NM_000142.4):c.1075+95C>G variant.

	Case 1	Case 2	Case 3	Case 4 (Described in This Report)
**Gender**	Male	Male	Male	Male
**Origin**	Chinese	Chinese	Chinese	European (France)
**Prenatal findings**	nd	Short lower limbs at third trimester ultrasound	nd	nd
**Age at the beginning of genetic investigation**	5 years	14 months	3 years	42 years
**Birth length**	nd	Normal (50 cm)	nd	Normal (48 cm)
**Limb aspect at birth**	nd	nd	nd	Micromelia
**Head circumference**	Mild macrocephaly	nd	Normal	Normal
**Face morphology**	Low nasal bridge	Bossing forehead, low nasal bridge, thick feet	Relatively normal facial features	Bossing forehead
**Intelligence**	Normal	Normal	Normal	Normal
**Other clinical features**	Shortening of limbs	Short limbs, continuous growth retardation	Short limbs (especially upper limbs), bowing legs	Shortening of limbs (rhizomelia and mesomelia), continuous growth retardation
**Radiologic findings**	Shortening and thickening of femora and tibia, metaphyseal flaring of distal femora and proximal tibia	Multiple skeletal abnormalities	Metaphyseal flaring of distal femora and proximal tibiae	Multiple spinal abnormalities
**Diagnosis**	Hypochondroplasia or mild achondroplasia	Achondroplasia or severe hypochondroplasia	Hypochondroplasia	Achondroplasia or severe hypochondroplasia

Cases 1, 2, and 3 were previously reported by Xu et al. [7]. Case 4 is the patient reported by this study. nd: not documented.

## Data Availability

The classification of the GRCh37-chr4:g.1805658C>G variant was submitted to MobiDetails [18] on 2 September 2022 and was reported in the Leiden Open Variation Database v.3.0 (LOVD^3^) [19] under the database ID (DB-ID) *FGFR3*_000225. The sequences of the primers used in this study are available on request.
